# Chronic Obstructive Pulmonary Disease as a Main Factor of Premature Aging

**DOI:** 10.3390/ijerph16040540

**Published:** 2019-02-13

**Authors:** Ilias Karametos, Paraskevi Tsiboli, Ilias Togousidis, Chrisi Hatzoglou, Grigorios Giamouzis, Konstantinos I. Gourgoulianis

**Affiliations:** 1 Internal Medicine Department, Hospital of Volos, 38221 Magnesia, Greece; 2 Biochemichal Laboratory Department, Hospital of Volos, 38221 Magnesia, Greece; biochemlab@ghv.gr (P.T.); togos@vol.forthnet.gr (I.T.); 3 Department of Medical Physiology, University of Thessaly Medical School, 41500 Larissa, Greece; chatz@med.uth.gr; 4 Department of Cardiology, University of Thessaly Medical School, 41110 Larissa, Greece; giamouzis@med.uth.gr; 5 Respiratory Medicine Department, University of Thessaly Medical School, University Hospital of Larisa, 41110 Larissa, Greece; kgourg@med.uth.gr

**Keywords:** chronic obstructive pulmonary disease, aging, biological marker, dehydroepiandrosterone, DHEA, growth hormone, GH

## Abstract

(1) Background: Chronic obstructive pulmonary disease (COPD) is defined as an inflammatory disorder that presents an increasingly prevalent health problem. Accelerated aging has been examined as a pathologic mechanism of many chronic diseases like COPD. We examined whether COPD is combined with accelerated aging, studying two hormones, dehydroepiandrosterone (DHEA) and growth hormone (GH), known to be characteristic biological markers of aging. (2) Methods: Data were collected from 119 participants, 70 (58.8%) COPD patients and 49 (41.2%) from a health control group over the period of 2014–2016 in a spirometry program. Information about their medical history, tobacco use, and blood tests was obtained. (3) Results: The average age of the health control patients was 73.5 years (SD = 5.5), and that of the COPD patients was 75.4 years (SD = 6.9). Both groups were similar in age and sex. A greater proportion of smokers were found in the COPD group (87.1%) versus the control group (36.7%). The majority of COPD patients were classified as STAGE II (51.4%) and STAGE III (37.1%) according to GOLD (Global Initiative for Chronic Obstructive Pulmonary Disease). Levels of DHEA (SD = 17.1) and GH (SD = 0.37) were significantly lower in the COPD group (*p* < 0.001) compared to those in the controls (SD = 26.3, SD = 0.79). DHEA and GH were more significant and negatively correlated with age. The regression equation of DHEA with age produced a coefficient equal to 1.26. In this study, the difference in DHEA between COPD patients and controls was, on average, 30.2 μg/dL, indicating that the biological age of a COPD patient is on average about 24 years older than that of a control subject of the same age. Similarly, the difference in GH between COPD patients and controls was, on average, 0.42 ng/mL, indicating that the biological age of a COPD patient is on average about 13.1 years older than that of a control subject of the same age. (4) Conclusions: The findings of our study strongly suggest the presence of premature biological aging in COPD patients. Their biological age could actually vary from 13 to 23 years older than non-COPD controls according to DHEA and GH variation.

## 1. Introduction

Chronic obstructive pulmonary disease (COPD) is an increasingly prevalent disorder of the respiratory system characterized by a progressive decline in lung function and chronic inflammatory response [1,2]. Its prevalence is high not only in elderly people, and it is expected to become the third leading cause of death in the world by 2020 [2,3].

In COPD, processes including oxidant/antioxidant, protease/antiprotease, and proliferative/antiproliferative balance, and the control of the inflammatory response become dysfunctional, as in aging [4,5]. A close relationship between the pathogenesis of COPD and aging has been reviewed, and an increase according to aging has been identified [6,7].

Aging is defined as a time-dependent progressive loss of physiological integrity, resulting in impaired function and increased vulnerability to death [8]. A recent attempt was made to describe the different metabolic and cellular markers of aging. In addition to what is meant by normal aging, many chronic diseases are dependent on age and encompass physiological mechanisms related to the aging process [9,10].

The contribution of aging markers has recently been reviewed in COPD patients [11]. This relationship was confirmed by “MARK-AGE”, a large-scale study that considered several physical parameters as “classical” candidates for aging biological markers, including lung function (as forced expiratory volume in 1 second (FEV1) and forced vital capacity (FVC)) together with immunological and systemic inflammation, and oxidative stress markers [12]. The major feature of aging is the role of hormones as key regulators of human muscle metabolism and physical function. Decline or even loss of sex hormones (androgens and estrogens) is combined with aging, which may be responsible for muscle weakness, muscle loss, decreased functional performance, and decreased life span [13].

The interconnection of the different markers of aging has not yet been studied in clinical subjects. We hypothesized that these markers, representing various interconnected aspects of the aging process as recently summarized, are altered in a cohort of COPD patients compared to the control group. These markers include dehydroepiandrosterone (DHEA)-S and growth hormone(GH)as a read-out of biological age.

The aim of the present study was to examine whether COPD is associated with accelerated aging using two hormones, dehydroepiandrosterone sulfate and growth hormone, representing biological markers of aging.

## 2. Materials and Methods

Participants were recruited in 20health centers of primary care in Thessaly, Greece, during a period of 24 months (January 2015 to December 2016). 

All volunteers were older than 40 years of age, residents near a primary healthcare practice, and were willing to participate in the spirometry program organized by the Respiratory Department of the University Hospital of Larisa. 

The study sample finally consisted of 70 newly diagnosed [14,15] patients with COPD and 48 non-COPD patients according to the spirometry test. Participants were excluded if they were unable to perform the spirometry test, if they had a history of respiratory tract infection in the past 4 weeks, had a history of hormone disorder or tumor disease, took hormone replacement treatment, or corticosteroid therapy.

The study was approved by the University of Thessaly Ethics Committee (6051/29-1-2014). All participants gave informed written consent and allowed the use of their personal data for research purposes.

### 2.1. Study Design

A study questionnaire was completed from all participants who were submitted to a physical examination. Personal data (age, marital status, education level, occupational exposure, rural residence) were assessed, including somatometric measurements such as body mass index (BMI), medical history of comorbidities [16], or any kind of drug treatment, history of recent or chronic symptoms of the respiratory system (i.e., cough, sputum production, dyspnea), and smoking habits. BMI was calculated as body weight divided by height squared (expressed in kg/m^2^).

Participants who had smoked over 100 cigarettes in their life were considered as smokers, and those who had given up smoking in the last 12 months as ex-smokers. Smoking status was measured by pack–years (PYS), defined as the number of cigarettes smoked per day divided by 20 and multiplied by years of smoking. According to their smoking habits, they were classified as never smokers, ex-smokers, and current smokers [17].

The study questionnaire was followed by tests, used in describing and giving information about the mental and physical status of the patients themselves, such as the COPD assessment test (CAT score), the clinical COPD questionnaire (CCQ), and the 12-Item short-form health survey (SF-12).

A morning blood-test sample was taken in order to examine the two hormones of aging, GH [18] and DHEA [19].

### 2.2. Spirometry

Spirometry was performed with a dry spirometer (Spirolab MIR-Italy) according to American Thoracic Society (ATS) recommendations [20]. Physicians with a special training program performed the spirometry test. Tests were repeated until three reproducible acceptable results were obtained, and the best FEV1, FVC, and FEV1/FVC ratio, were recorded [20]. A bronchodilator reversibility test using 400mcg of salbutamol was performed on all patients with obstructive spirometry findings. Obstructive spirometry was defined as an FEV1/FVC ratio of <0.7 in accordance with GOLD guidelines [1]. An increase in FEV_1_>12% and >200 ml from the baseline was considered reversible [21].

### 2.3. COPD Diagnosis

All participants were examined by chest physicians who established the diagnosis of COPD. All patients with a previous diagnosis of COPD were again evaluated by physicians [22] and current spirometry confirmed the diagnosis and classified the patient according to GOLD stages. A previous diagnosis of COPD was based on received medication and the patients’ medical records [23]. Classification of COPD was based on post bronchodilator FEV1, according to the GOLD guidelines (Stage I—mild COPD, FEV1 >80.0% predicted; Stage II—moderate COPD, 50.0% ≤ FEV1 < 80.0% predicted; Stage III—severe COPD, 30.0% ≤ FEV1 < 50.0%; Stage IV—very severe COPD, 30.0% ≤ FEV1 or FEV1 < 50% predicted with respiratory failure) [24].

### 2.4. Laboratory Tests

Blood test was drawn from participants in the morning time only and blood was drawn into two 10 mL vacutainers. All blood was immediately spun and aliquoted, and the serum was stored at −80 °C until assays were performed. Serum levels of DHEA–sulfate (DHEA-S) and GH were measured in a solid-phase, two-site chemiluminescent immunometric assay according to the manufacturer’s instructions. The analysis system of the serum was the Immulite 2000 immunoassay(Siemens AG, Berlin, Germany) with a coefficient of variation of less than 5.23%. Theused method was the microparticular enzyme immunoassay (MPEIA).Control and calculation were held before testing.

The laboratory assay range (20–80 percentile) for participants aged 15–80 years was 100–290 μg/dL for DHEA, and 3–8 ng/mL for GH, and was dependent on age, with higher values at younger ages [25].

### 2.5. Statistic Analysis

Quantitative variables are expressed as mean values (standard deviation (SD)). Qualitative variables are expressed as absolute and relative frequencies. For the comparison of proportions, chi-square and Fisher’s exact tests were used. Pearson’s correlation coefficients (r) were used to test the association of two continuous measures. Student’s t-tests were used for the comparison of continuous variables between two groups, analysis of variance (ANOVA) was used for the comparison of continuous variables between more than two groups, and Bonferroni correction was used in order to control for type I error, where the level of significance was set at 0.05/No of comparisons. Mean differences along with their standard errors (SE) were also reported. Multiple linear regression analysis was used to find independently associated factors with DHEA and DH. Regression coefficients that reflected the change in the value of dependent variables corresponding to the unit change in independent variable β, along with SE, were computed from the results of the linear regression analyses. All *p*-values reported are two-tailed. Statistical significance was set at 0.05, and analyses were conducted using SPSS statistical software (version 19.0).

## 3. Results

**Figure 1:**
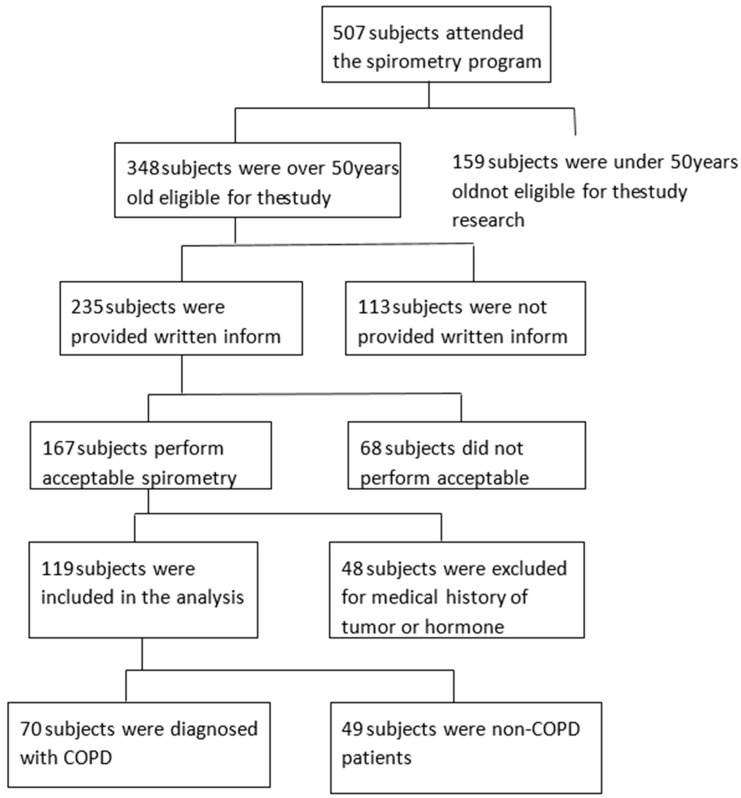
Flowchart of the study selection process. Notes: Exclusion criteria: No smoking history, age under 50 years old, a history of tumor disease or hormone replacement, or not capable of performing spirometry. Excluded subjects: 159 subjects aged <50 years, 48 subjects with hormone replacement and tumor history, and 68 subjects who did not achieve the spirometry procedure.

The flowchart of the study participants is presented in Figure 1.

During the two years of the study, 507 participants attended the spirometry program. Among them, 168 participants (33.13%) were able to correctly perform the spirometry test, 48 were diagnosed with hormone disorder, and only 119 (23.47%) from the whole sample were finally included in the study.

The sample consisted of 119 participants, 70 of whom were originally diagnosed with COPD and 49 without COPD (control group), with a mean age of 74.5 years (SD = 6.5).

Demographics and clinical characteristics of the two study groups are presented in Table 1. 

The groups consisted of rural residents, similar in age, sex, BMI, family history of COPD, when they started smoking, pack–years, presence of hypertension, arrhythmia, diabetes, allergies, depression, and previous surgeries. Both groups were overweight according to their BMI.

A greater proportion of smokers was found in the COPD group. 

COPD patients were newly diagnosed for the first time in a proportion of 70% men, 87.5% current smokers, with 77 PYS (number of cigarettes smoked per day divided by 20 and multiplied by years of smoking) already being in 51.4% STAGE II of classification of COPD.

Dyslipidemia (41.4%) and cardiovascular disease (41.4%) were also more frequent in the COPD group. There was no significant difference in the presence of hypertension between groups.

COPD patients were more often male, overweight, older, current or ex-heavy smokers. All respiratory symptoms (cough, sputum, dyspnea) were more common in COPD patients.

**Figure 2:**
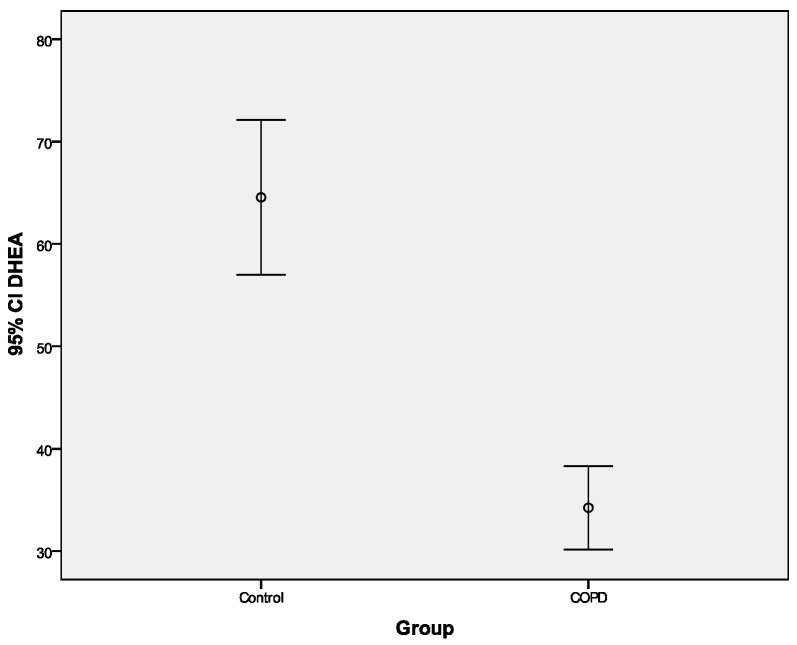
Mean DHEA values in the control and chronic obstructive pulmonary disease (COPD) group.

**Figure 3:**
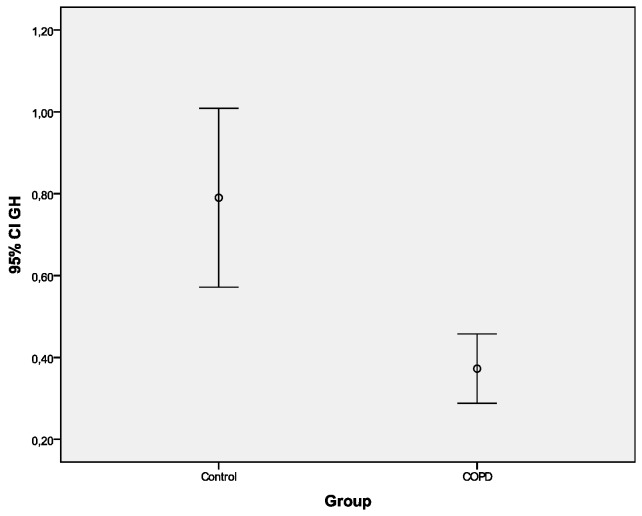
Mean GH values in the control and COPD group.

Univariate analysis for DHEA and GH: Levels of DHEA (mean difference: 30.2 (SE = 9.0)), and GH (mean difference: 0.42 (SE = 0.16)) were significantly lower in the COPD group compared with those in the controls (*p* < 0.001 Student’s t-test) (Table 2, Figure 2 and Figure 3). Additionally, analysis revealed that DHEA and GH levels were different between the COPD patients, nonsmoker controls and smoker controls. Specifically, post hoc comparisons after Bonferroni correction showed that COPD patients had significantly lower DHEA and GH levels when compared with both nonsmoker controls (*p* < 0.001 and *p* = 0.019, for DHEA and GH, respectively)and smoker controls (*p* < 0.001 and *p* = 0.001, for DHEA and GH, respectively).

As expected, CAT, MRC breathless scale, and CCQ had greater scores in the COPD group, while SF-12 dimensions (See Appendix A) had significantly lower scores in the COPD group (*p* < 0.001 Student’s t-test). DHEA and GH were positively correlated with FEV1ml (*p* < 0.001), FEV1% (*p* < 0.001), and FEV1/FVC (*p* < 0.001). No gender differences were found for DHEA and GH, but both DHEA and GH were significantly and negatively correlated with age. Further more, DHEA levels were lower in smokers. Both DHEA and GH were significantly and negatively correlated with the COPD assessment test (CAT), the MRC breathlessness scale, and all clinical COPD questionnaire (CCQ) dimensions (Table 3).

**Figure 4:**
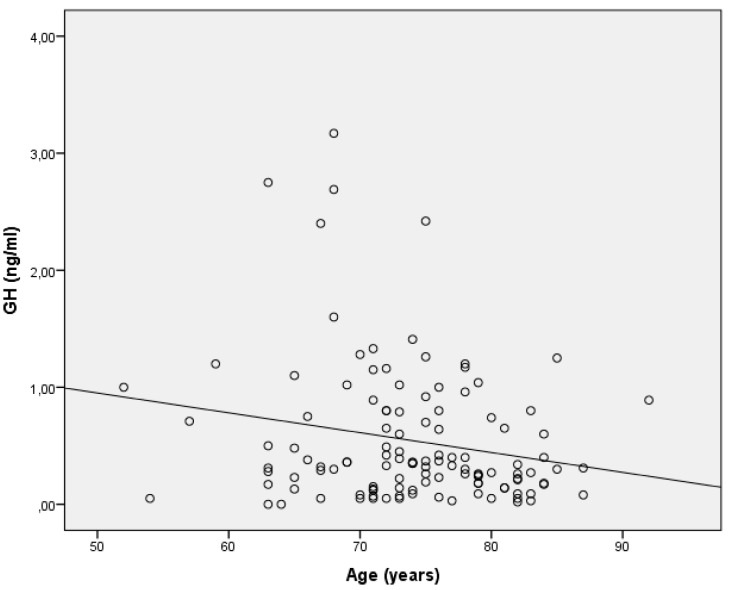
GH decline with age.

**Figure 5:**
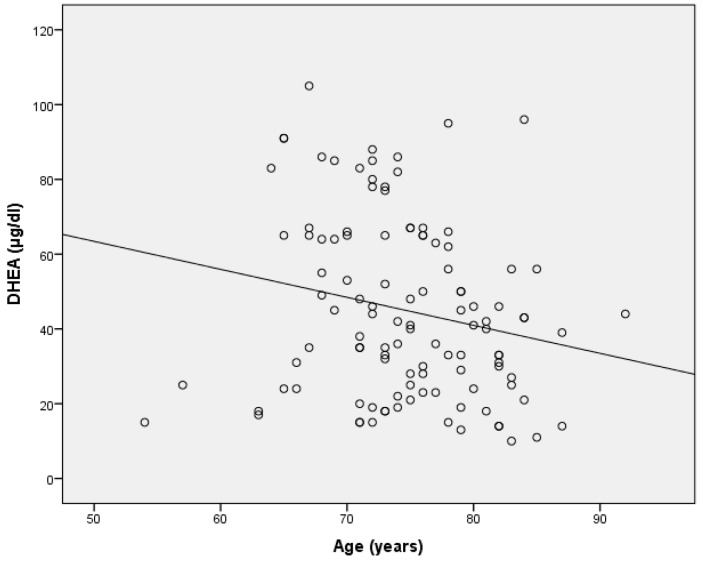
DHEA decline with age.

The regression equations of DHEA and GH with age indicated that one-year age increase was associated with a 1.26 units decrease in DHEA and 0.032 units decrease in GH.In the present study, the difference in DHEA between COPD patients and controls was, on average, 30.2 μg/dL, indicating that the biological age of a COPD patient is, on average, about 30.2/1.26 = 24, that is, 24 years older than the age of a control subject of the same age. Similarly, the difference in GH between COPD patients and controls was, on average, 0.42 ng/mL, indicating that the biological age of a COPD patient is, on average, about 0.42/0.032 = 13.1, that is, 13.1 years older than that of a control subject of the same age (Figure 4 and Figure 5). 

Multiple regression analysis was conducted with DHEA and GH as the dependent variables. The independent variables that were entered in the model were sex, age, smoking, CAT, and BMI. Concerning DHEA, the factors that were significant in the regression model were CAT (*β* = −1.19 μg/dL, SE = 0.24, *p* < 0.001) and age (*β* = −0.61 μg/dL, SE = 0.30, *p* = 0.032),indicating that greater levels of CAT and greater age were associated with lower levels of DHEA. For GH, the significant factors were CAT (*β* = −0.015 ng/mL, SE = 0.006, *p* = 0.018) and age (*β* = −0.017 ng/mL, SE = 0.008, *p* = 0.036), indicating that increased levels of CAT and age were associated with lower levels of GH.

## 4. Discussion

In our study, the premature biological aging of COPD patients was revealed, as it is expressed by both DHEA and GH. Dehydroepiandrosterone and growth hormone alike were found to be significantly influenced in these patients. Even though COPD has been incriminated for premature aging, as far as we are aware, this is the only study to have assessed the correlation between COPD, DHEA, and GH.

It is widely known that changes in the endocrine system are related to age, particularly those in sex hormones. It is common knowledge that there is a gradual decline in circulating testosterone concentrations due to aging [26,27]. This phenomenon is attributed to decreased hormone production. It has been estimated that starting at the age of 35–40 years, circulating testosterone levels decrease by approximately 1–3% per year [27]. This correlation between DHEA and age was also confirmed by the findings of the present study. Dehydroepiandrosterone-S levels showed significant decline correlated with age for both sexes. Furthermore, DHEA-S was found to be significantly correlated with various parameters of pulmonary function, including MRC, FEV1%, and blood 0_2_ saturation. Consequently, as it might have been expected, DHEA-S showed significantly lower values in COPD patients. Additionally, DHEA-S levels were lower in smokers compared to those in the control group, but significantly higher than those in COPD patients, evidence that must be taken into consideration.

On the other hand, growth-hormone levels are higher in early life, corresponding to the period of rapid somatic growth [28]. They start to decline soon after achieving adult body size and the completion of physical and reproductive maturation. This process continues during adult life. Consequently, plasma GH levels are significantly lower in the elderly [28]. This correlation was also confirmed in the present study, with GH showing significantly negative correlation with age. 

Furthermore, GH like DHEA-S was significantly correlated with various pulmonary parameters, including FEV1%, FVC, CAT score, and 0_2_ saturation. Significantly lower GH levels were also revealed in COPD patients. 

The present study has focused on sex hormones and the growth hormone. Longitudinal studies have shown strong evidence that testosterone, estrogen, DHEAS, and growth hormone IGF-1 are linked with the risk of premature mortality and physical frailty [29]. 

DHEAS was used as an endocrine marker of aging in calorie-restriction studies, concluding that it can reliably predict aging in animals [30].

Other markers have been also proposed in assessing biological age, including telomere, expression of epigenetic alterations such as DNA repair proteins (Ku70/80 and TERF2) and markers of cellular senescence (p16/21), and anti-aging molecules (sirtruin 1, total (T) and soluble (S) klotho) [31]. Interestingly, telomere length was significantly shorter in the COPD group compared to that in the control group even though multiple corrections were held. This indicates that COPD can serve as a model for accelerated biological aging by this marker, as proposed [32,33]. Researchers observed that the difference in telomere length between patients and controls was, on average, around300 bp, indicating that the biological age of a COPD patient is, on average, about 300/40 = 7.5 years older than that of a control subject of the same age.

Another aging theory for COPD focuses on the involvement of sirtruin(SIRT1) in the regulation of inflammation and premature senescence, all crucial characteristics of COPD phenotypes. Findings contributed to the hypothesis that COPD could be considered a disease of accelerated aging and underline the potential of SIRT1 as a valid therapeutic target to treat respiratory disorders sharing chronic inflammation [34].

Kazuhiro Ito et al., in their analysis about geroprotectors, concluded that COPD is one of the most prevalent chronic inflammatory diseases in world populations. Loss of the working generation and disabilities associated with COPD should require urgent awareness from governments, physicians, and scientists, and a new treatment strategy should be considered, as current therapies are not useful in stopping the progression of COPD. The role of accelerated aging in COPD progression is now strongly supported by a number of recent studies. Geroprotection or anti-aging therapy is a novel and attractive strategy to treat age-associated inflammatory diseases or to increase the quality of life of elderly patients. For this purpose, geroprotectors are not used to extend the lifespan, but to prevent premature aging of the lungs [35].

In the present study, it was observed that 36% of newly diagnosed patients were already Stage II and 26% Stage III, which is supported by previous studies [36]. It could be strongly argued that, due to the production of constant stresses that induce cell damage and eventual senescence, COPD might be directly responsible for accelerated aging, with all its untoward effects, rather than being a consequence of aging [37].

In another clinical study, functional performance and cognitive status were compared in COPD patients of different ages. Significant reductions in functional capacity, cognitive assessment, and lower-limb muscle force, as well as increased inflammatory markers, were observed in the older group [38].

Over the last 50 years [39,40], there have been efforts to develop markers of aging, but the complexity of the aging phenotype [41] leads to conceptual and practical difficulties. Beyond earlier efforts [42], there is currently no accepted definition of aging biomarkers or criteria for their selection, which has resulted in a lack of validated tools for assessing healthy aging. 

Both DHEA-S and GH were selected among well-established markers, with evidence supporting a strong association with aging phenotypes. They are cost-effective and practical for use in larger-scale studies, or even in everyday practice implementation.

For some biological markers, the relationship with aging appears to be nonlinear; for example, both high and low IGF-1 are related with greater mortality rates. DHEA-S declines with age from the third decade, and low DHEA-S is associated with increased mortality in older subjects with concurrent frailty. Hormone replacement studies suggest use for both testosterone and estrogen and risk physical frailty and bone health [43,44].

The present study revealed that the use of biological markers commonly used in a range of settings, like DHEA and GH, could provide valuable data in discriminating between healthy and premature aging regarding COPD patients.

Concrete evidence is needed to enhance the understanding of the relationships between cortisol, DHEAS, DHEA-S ratio, adipokines (adiponectin, leptin, ghrelin), somatostatin with aging, frailty, and mortality [29].

Overall current knowledge in the molecular mechanisms of lung aging is limited. Studies, such as the one by de Vries, are important if we are to understand how molecular aging mechanisms could be minimized to improve the quality of life of patients with obstructive lung diseases such as COPD [45]. Keene et al., in their research about biomarkers, predictive of exacerbations in COPD, concluded that there was poor reproducibility of specific biomarkers [46].

## 5. Limitations

The present study is limited by its cross-sectional design. Nevertheless, current findings are significant, revealing a new accessible path in approaching premature aging in COPD patients. Prospective studies are needed in the future to verify these findings. Even though507 individuals attended the spirometry program, only 119 participants were finally eligible. It is possible that a greater number would have better determined the observed differences in the two measured hormones. Nevertheless, the actual number of eligible participants was proven to be adequate to reveal significant differences between COPD patients and the control group. The starting age of participants should also be lower in future projects. That would allow for a lower starting chronological and biological age of COPD patients in order to support a prospective design. More age-related biomarkers could also be calculated according to somatometric characteristics, hormones, inflammatory markers, and, on the molecular level, sirtruins, telomeres, and genes.

## 6. Conclusions

In summary, our findings concluded that COPD patients present reduced DHEA-S and GH levels. Taking into consideration the correlation of these two hormones as biological markers of age, based on the current findings, it is suggested that a COPD patient is 24 years older as far as DHEA is concerned and 13 years older with regard to GH. This is an exceptional finding regarding the extent to which COPD influences premature aging. Furthermore, additional research is needed toreveal accurate aging biomarkers that can easilybe assessed.

## Figures and Tables

**Table 1:** Demographics and clinical characteristics of study participants (N = 119).

	Group		*P*
	Control	COPD	
	N (%)	N (%)	
**Sex**			
Men	28 (57.1)	49 (70.0)	0.149 ^+^
Women	21 (42.9)	21 (30.0)	
Age, mean (SD)	73.5 (5.5)	75.4 (6.9)	0.114 ^‡^
BMI, mean (SD)	27.2 (4.2)	27.1 (4.1)	0.867 ^‡^
**BMI**			
Normal	14 (28.6)	20 (28.6)	0.773 ^+^
Overweight	25 (51.0)	32 (45.7)	
Obese	10 (20.4)	18 (25.7)	
Family COPD history	2 (4.1)	8 (11.4)	0.194 ^++^
Other family history	5 (10.2)	8 (11.4)	0.833 ^+^
**Smoking**			
No	31 (63.3)	9 (12.9)	<0.001 ^+^
Yes	18 (36.7)	61 (87.1)	
Age at start of smoking, mean (SD)	19.9 (3.1)	18.3 (4.0)	0.100 ^‡^
Age at stop of smoking, mean (SD)	57.9 (9.7)	62.6 (9.9)	0.084 ^‡^
Packet–years, mean (SD)	63.5 (35.3)	77.1 (37)	0.141 ^‡^
**Stage**			
I	-	6 (8.6)	-
II	-	36 (51.4)	-
III	-	26 (37.1)	-
IV	-	2 (2.9)	-
Hypertension during examination	19 (38.8)	28 (40)	0.893 ^+^
Hypertension	30 (61.2)	43 (61.4)	0.982 ^+^
Dyslipidemia	11 (22.4)	29 (41.4)	0.031 ^+^
Arrhythmia	10 (20.4)	23 (32.9)	0.135 ^+^
Cardiovascular disease	9 (18.4)	29 (41.4)	0.008 ^+^
Diabetes	16 (32.7)	27 (38.6)	0.508 ^+^
Thyroid-gland disease	8 (16.3)	5 (7.1)	0.114 ^+^
Gastroenteric disorders	6 (12.2)	14 (20.3)	0.251 ^+^
Leaver disease	2 (4.1)	1 (1.4)	0.569 ^++^
Allergy	5 (10.2)	2 (2.9)	0.123 ^++^
Depression	3 (6.1)	5 (7.1)	1.000 ^++^
Surgery	13 (26.5)	14 (20)	0.403 ^+^
Other disease	14 (28.6)	15 (21.4)	0.372 ^+^

^+^ Pearson’s chi-square; ^++^ Fisher’s exact test; ^‡^ Student’s t-test Data areexpressedas mean ± standard deviation or as frequency (percentage).

**Table 2:** Dehydroepiadrosterone(DHEA) and Growth Hormone (GH) levels in the study groups.

	DHEA		GH	
Group	Mean (SD)	*P*	Mean (SD)	*P*
Control	64.6 (26.3)	<0.001 ^+^	0.79 (0.76)	<0.001 ^+^
COPD	34.2 (17.1)		0.37 (0.36)	
Nonsmoker controls	64.77 (29.91)	<0.001 ^++^	0.71 (0.61)	<0.001 ^++^
Smoker controls	64.17 (19.44)		0.94 (0.96)	
COPD	34.23 (17.09)		0.37 (0.36)	

^+^ Student’s t-test; ^++^ ANOVA.Data areexpressedas mean ± standard deviation or as frequency (percentage). (Significantly lower DHEA and GH among control and COPD group, not in smoker group).

**Table 3:** Association of DHEA and GH with demographic dyspnea indices and quality of life dimensions.

	DHEA		GH	
	Mean (SD)	P	Mean (SD)	P
Sex				
Men	45.25 (22.29)	0.407 ^++^	0.53 (0.57)	0.761 ^++^
Women	49.4 (31.89)		0.57 (0.65)	
Age, r ^+^	−0.20	0.031	−0.20	0.027
Smoking				
No	56.5 (31.07)	0.003 ^++^	0.64 (0.58)	0.229 ^++^
Yes	41.76 (21.63)		0.5 (0.6)	
CAT, r ^+^	−0.48	<0.001	−0.27	0.003
MRC, r ^+^	−0.48	<0.001	−0.26	0.004
CCQ, r ^+^	−0.48	<0.001	−0.28	0.002
CCQ–SYMPTOM, r ^+^	−0.49	<0.001	−0.29	0.002

^+^ Pearson’s correlation coefficient; ^++^ Student’s t-test.

## Abbreviations

COPD	Chronic Obstructive Pulmonary Disease
DHEA-S	Dihydroepiandrosterone Sulfate
GH	growth hormone; SIRT:sirtruin
IGF-1	insulin growth factor -1
FEV1	forced expiratory volume
FVC	forced vital capacity
CAT	chronic obstructive pulmonary disease assessment test
MRC	breathless scale
CCQ	chronic obstructive pulmonary disease clinical questionnaire

## Appendix A

**Questionnaire A1. SF-12^®^Patient Questionnaire**

Patient Initials________________Date of Birth:____/____/____Patkey:______

Examination Period: _____Preop (1)_____3 Year (4)_____Immediate Postop (2)_____5 Year (5)_____1 Year (3)_____Other (specify) (6):______________

SF-12^®^: This information will help your doctors keep track of how you feel and how well you are able to do your usual activities. Answer every question by placing a check mark on the line in front of the appropriate answer. It is not specific for arthritis. If you are unsure about how to answer a question, please give the best answer you can and make a written comment beside your answer.

1. In general, would you say your health is:_____Excellent (1)_____Very Good (2)_____Good (3)_____Fair (4)_____Poor (5)

The following two questions are about activities you might do during a typical day.Does YOUR HEALTH NOW LIMIT YOU in these activities? If so, how much?

2. MODERATE ACTIVITIES, such as moving a table, using a vacuum cleaner, bowling, or playing golf: _____Yes, Limited A Lot (1) _____Yes, Limited A Little (2) _____No, Not Limited At All (3)

3. Climbing SEVERAL flights of stairs: _____Yes, Limited A Lot (1) _____Yes, Limited A Little (2) _____No, Not Limited At All (3)

During the PAST 4 WEEKS, have you had any of the following problems with your work or other regular activities AS A RESULT OF YOUR PHYSICAL HEALTH?

4. ACCOMPLISHED LESS than you would like: _____Yes (1) _____No (2)

5. Were limited in the KIND of work or other activities: _____Yes (1) _____No (2)

Doctor’s Initials __________ Date:_____________
